# Supplementary material for: Maternal-fetal immune responses in pregnant women infected with SARS-CoV-2
Source: Nat Commun. 2022 Jan 18;13:320. doi: 10.1038/s41467-021-27745-z (PMC8766450; doi:10.1038/s41467-021-27745-z)
Supplement: Supplementary file 1 — Supplementary Information [file 41467_2021_27745_MOESM1_ESM.pdf]

Supplementary Information

Maternal-Fetal Immune Responses in Pregnant Women Infected with SARS-CoV-2

Valeria Garcia-Flores[1,2], Roberto Romero[1,3-6,*], Yi Xu[1,2], Kevin Theis[1,2,7], Marcia Arenas-Hernandez[1,2], Derek Miller[1,2], Azam Peyvandipour[1,2,5], Gaurav Bhatti[1,2], Jose Galaz[1,2], Meyer Gershater[1,2], Dustyn Levenson[1,2], Errile Pusod[1,2], Li Tao[1,2], David Kracht[1,2], Violetta Florova[1,2], Yaozhu Leng[1,2], Kenichiro Motomura[1,2], Robert Para[1,2], Megan Faucett[1,2], Chaur-Dong Hsu[1,2,8], Gary Zhang[5], Adi L. Tarca[1,2,9], Roger Pique-Regi[1,2,5,*], Nardhy Gomez-Lopez[1,2,8,*]

[1]Perinatology Research Branch, Division of Obstetrics and Maternal-Fetal Medicine, Division of Intramural Research, *Eunice Kennedy Shriver* National Institute of Child Health and Human Development, National Institutes of Health, U.S. Department of Health and Human Services (NICHD/NIH/DHHS); Bethesda, Maryland, 20892 and Detroit, Michigan, 48201, USA;

[2]Department of Obstetrics and Gynecology, Wayne State University School of Medicine, Detroit, Michigan, 48201, USA;

[3]Department of Obstetrics and Gynecology, University of Michigan, Ann Arbor, Michigan, 48109, USA;

[4]Department of Epidemiology and Biostatistics, Michigan State University, East Lansing, Michigan, 48824, USA;

[5]Center for Molecular Medicine and Genetics, Wayne State University, Detroit, Michigan, 48201, USA;

[6]Detroit Medical Center, Detroit, Michigan, 48201, USA;

[7]Department of Biochemistry, Microbiology and Immunology, Wayne State University School of Medicine, Detroit, Michigan, 48201, USA;

[8]Department of Physiology, Wayne State University School of Medicine, Detroit, Michigan 48201, USA;

[9]Department of Computer Science, Wayne State University College of Engineering, Detroit, Michigan, 48201, USA

*Corresponding authors:

nardhy.gomez-lopez@wayne.edu

rpique@wayne.edu

prbchiefstaff@med.wayne.edu

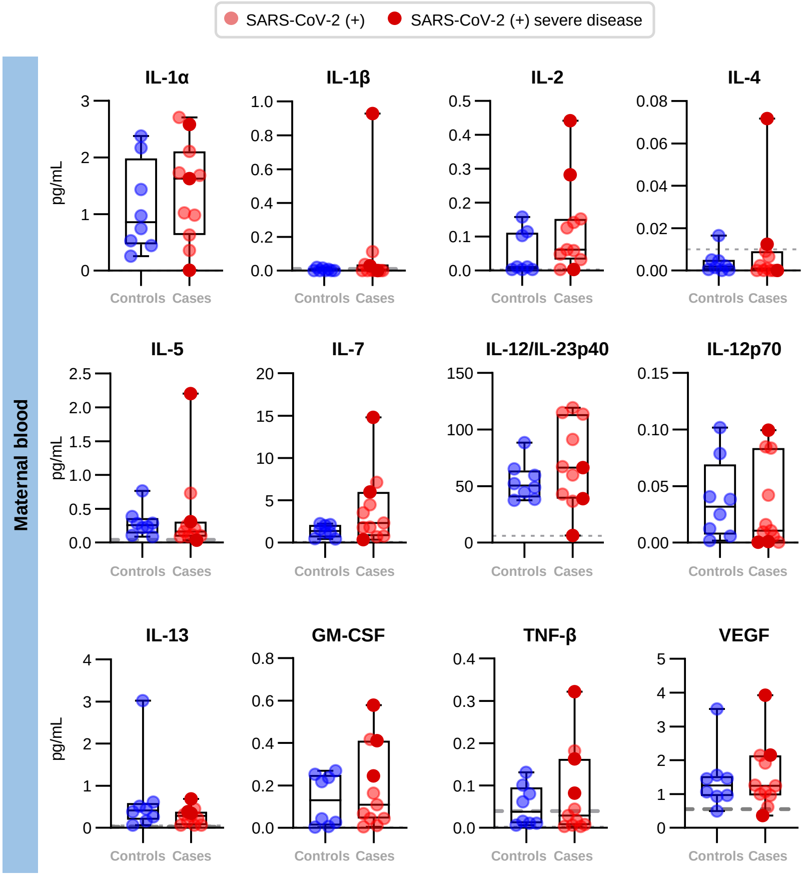

Supplementary Figure 1. Cytokine concentrations in the peripheral blood of pregnant women with SARS-CoV-2 infection. Plasma concentrations of IL-1α, IL-1β, IL-2, IL-4, IL-5, IL-7, IL-12/IL-23p40, IL-12p70, IL-13, GM-CSF, TNF-β, and VEGF from the maternal blood of SARS-CoV-2 (+) (n = 11) or control (n = 8) women. Data are shown as boxplots where midlines indicate medians, boxes indicate interquartile range, and whiskers indicate minimum/maximum range. Blue dots indicate control women, light red dots indicate SARS-CoV-2 (+) women, and dark red dots indicate women with severe COVID-19. Gray dotted lines indicate the lower limit of detection. Differences between groups were evaluated by using linear mixed-effect models. Significant differences are based on $p < 0.05$.

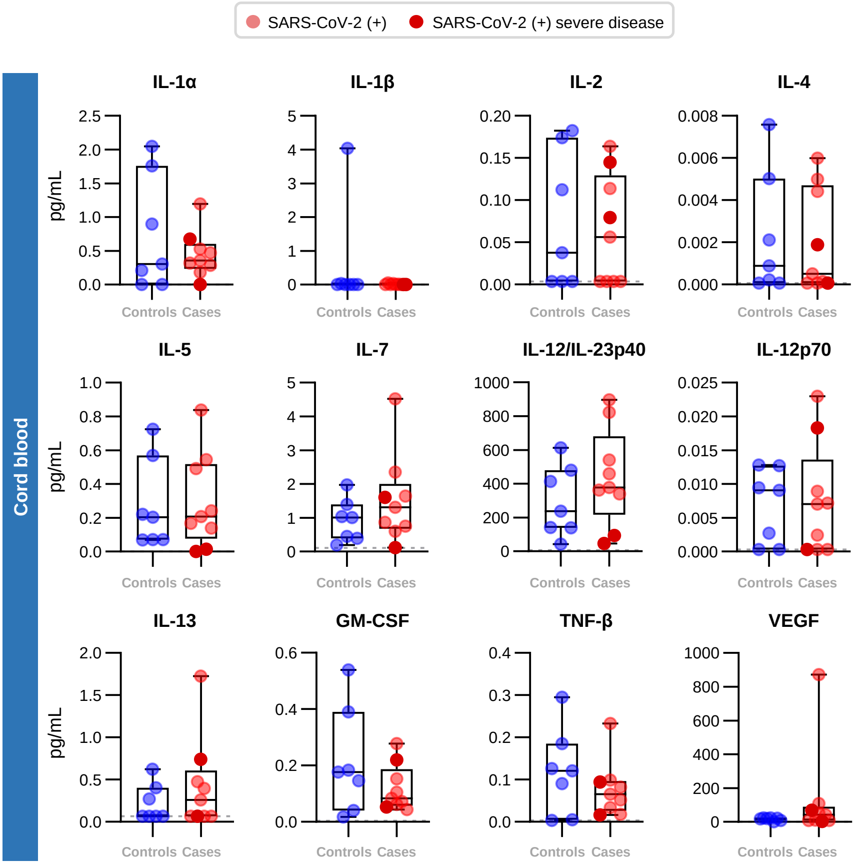

Supplementary Figure 2. Cytokine concentrations in the cord blood of neonates born to women with SARS-CoV-2 infection. Plasma concentrations of IL-1α, IL-1β, IL-2, IL-4, IL-5, IL-7, IL-12/IL-23p40, IL-12p70, IL-13, GM-CSF, TNF-β, and VEGF from the cord blood of neonates born to SARS-CoV-2 (+) (n = 9) or control women (n = 7). Data are shown as boxplots where midlines indicate medians, boxes indicate interquartile range, and whiskers indicate minimum/maximum range. Blue dots indicate control women, light red dots indicate SARS-CoV-2 (+) women, and dark red dots indicate women with severe COVID-19. Gray dotted lines indicate the lower limit of detection. Differences between groups were evaluated by linear mixed-effect models. Significant differences are based on $p < 0.05$.

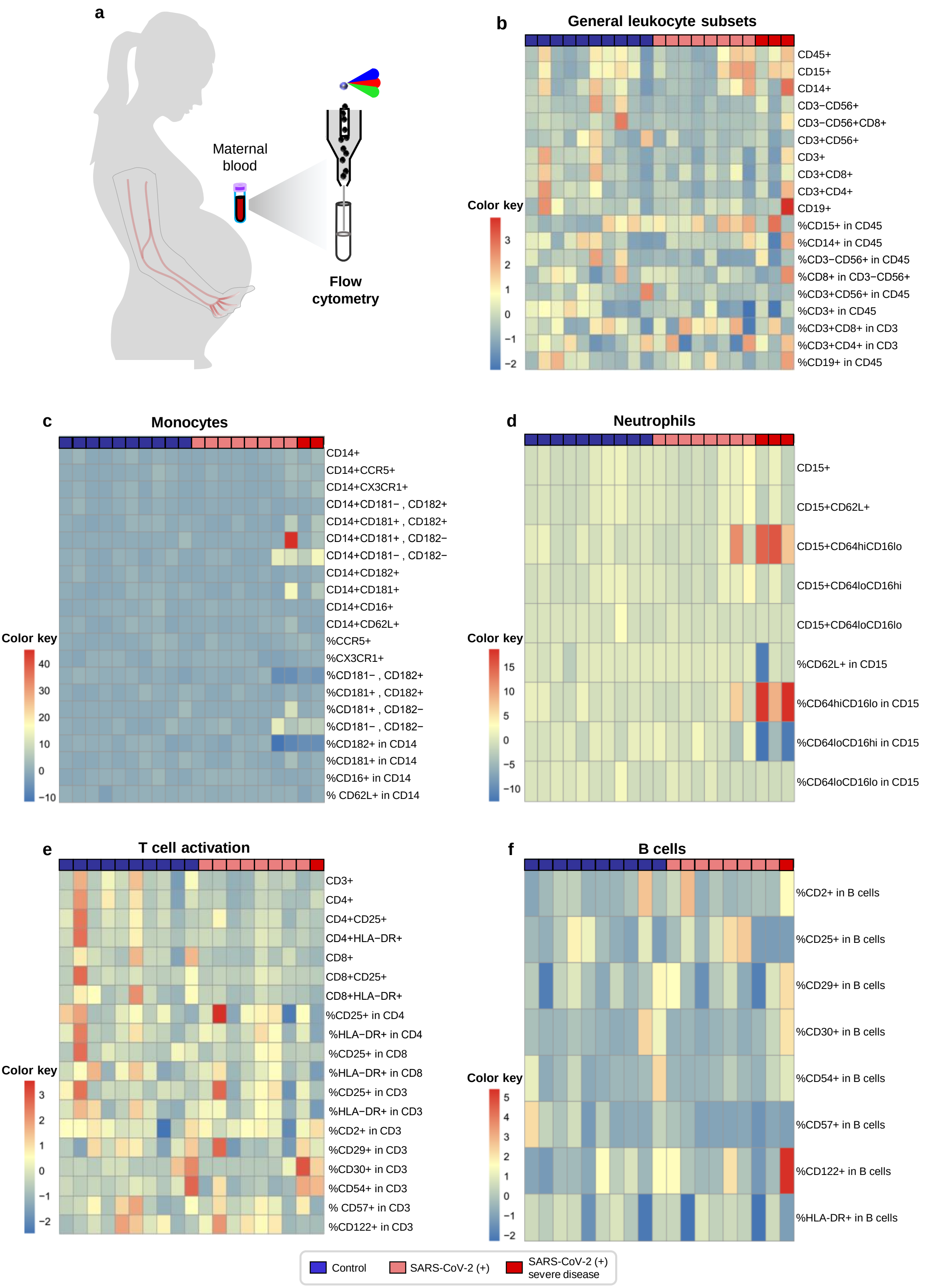

Supplementary Figure 3. Immunophenotyping of leukocyte subsets in the maternal blood of women with SARS-CoV-2 infection. (a) Peripheral blood was collected from SARS-CoV-2 (+) or control women (n = 9-11 per group) for immunophenotyping. Representative heat maps showing the abundance (z-scores) of **(b)** general leukocyte subsets, **(c)** monocyte subsets, **(d)** neutrophil subsets, **(e)** activated T-cell subsets, and **(f)** B cell subsets. Abundances are calculated using z-scores from the peripheral blood of SARS-CoV-2 (+) and control women. Blue squares indicate control women, light red squares indicate SARS-CoV-2 (+) women, and dark red squares indicate women with severe COVID-19. Red and blue indicate increased and decreased abundance, respectively.

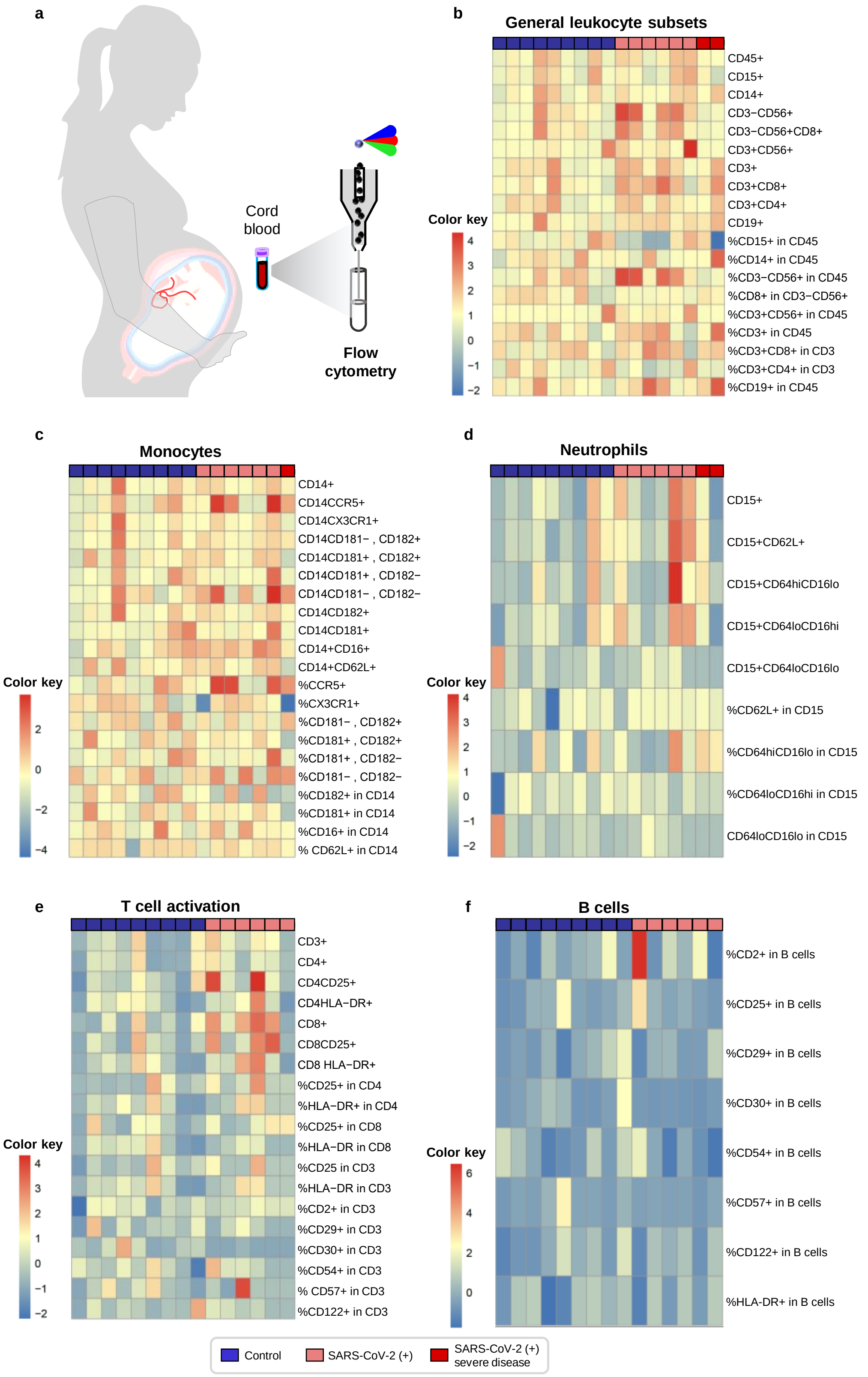

Supplementary Figure 4. Immunophenotyping of leukocyte subsets in the cord blood of neonates born to women with SARS-CoV-2 infection. (a) Cord blood was collected from neonates born to SARS-CoV-2 (+) or control women (n = 9-11 per group) for immunophenotyping. Representative heat maps showing the abundance (z-scores) of **(b)** general leukocyte subsets, **(c)** monocyte subsets, **(d)** neutrophil subsets, **(e)** activated T-cell subsets, and **(f)** B cell subsets. Abundances are calculated using z-scores from the cord blood of neonates born to SARS-CoV-2 (+) and control women. Blue squares indicate control women, light red squares indicate SARS-CoV-2 (+) women, and dark red squares indicate women with severe COVID-19. Red and blue indicate increased and decreased abundance, respectively.

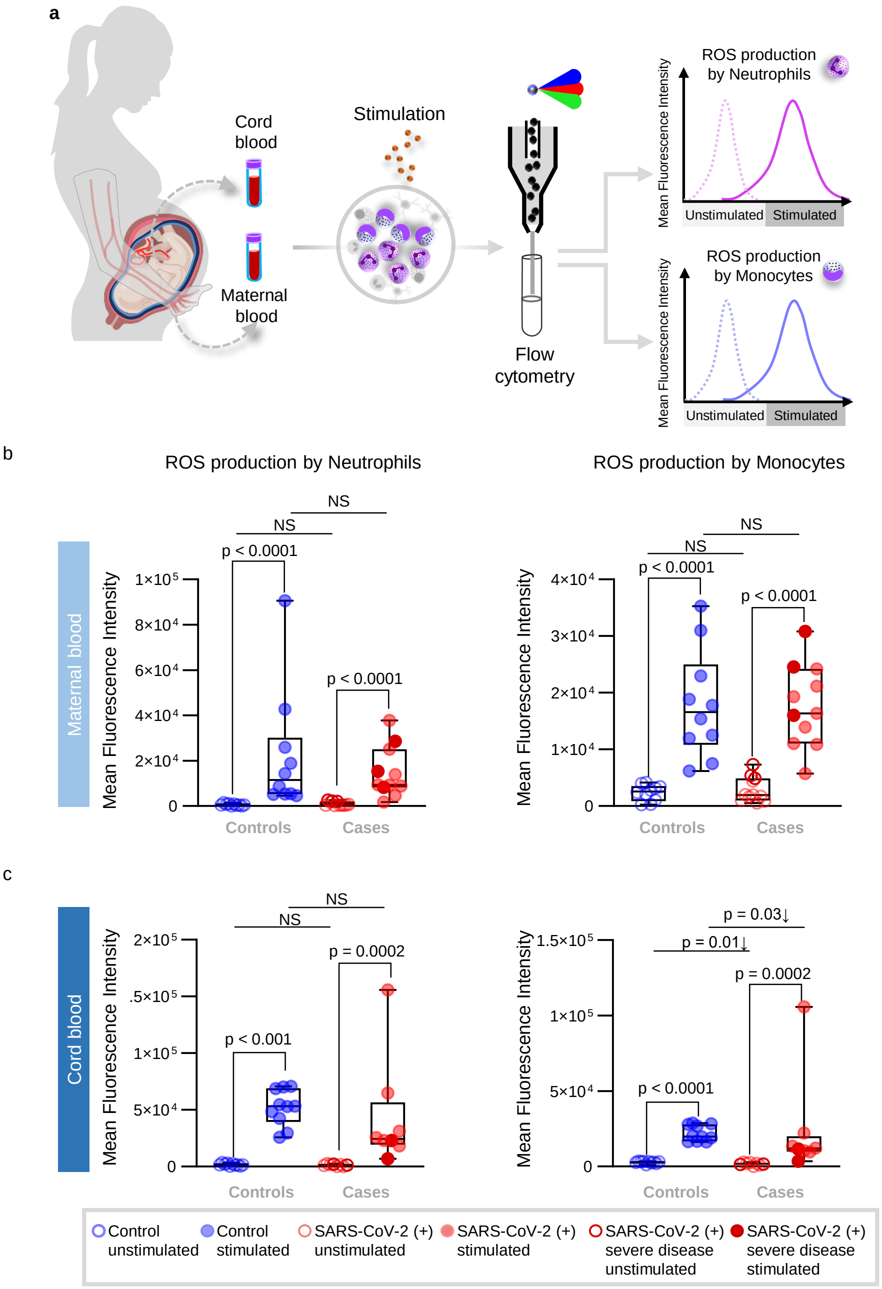

Supplementary Figure 5. Reactive oxygen species production by neutrophils and monocytes in maternal and cord blood of women with SARS-CoV-2 infection and their neonates. (a) Maternal blood [n = 10 control, 11 SARS-CoV-2 (+)] and cord blood [n = 10 control, 8 SARS-CoV-2 (+)] were collected and leukocytes were stimulated *in vitro* prior to the evaluation of reactive oxygen species (ROS) production by neutrophils and monocytes. ROS production by neutrophils and monocytes in **(b)** maternal peripheral blood and **(c)** cord blood with or without stimulation as represented by mean fluorescence intensity (MFI). Data are shown as boxplots where midlines indicate medians, boxes indicate interquartile range, and whiskers indicate minimum/maximum range. Differences between groups were evaluated by two-sided Mann-Whitney U-tests. P-values are considered significant when $p < 0.05$. Open dots indicate unstimulated cells and filled dots indicate stimulated cells. Blue dots indicate control women, light red dots indicate SARS-CoV-2 (+) women, and dark red dots indicate women with severe COVID-19.

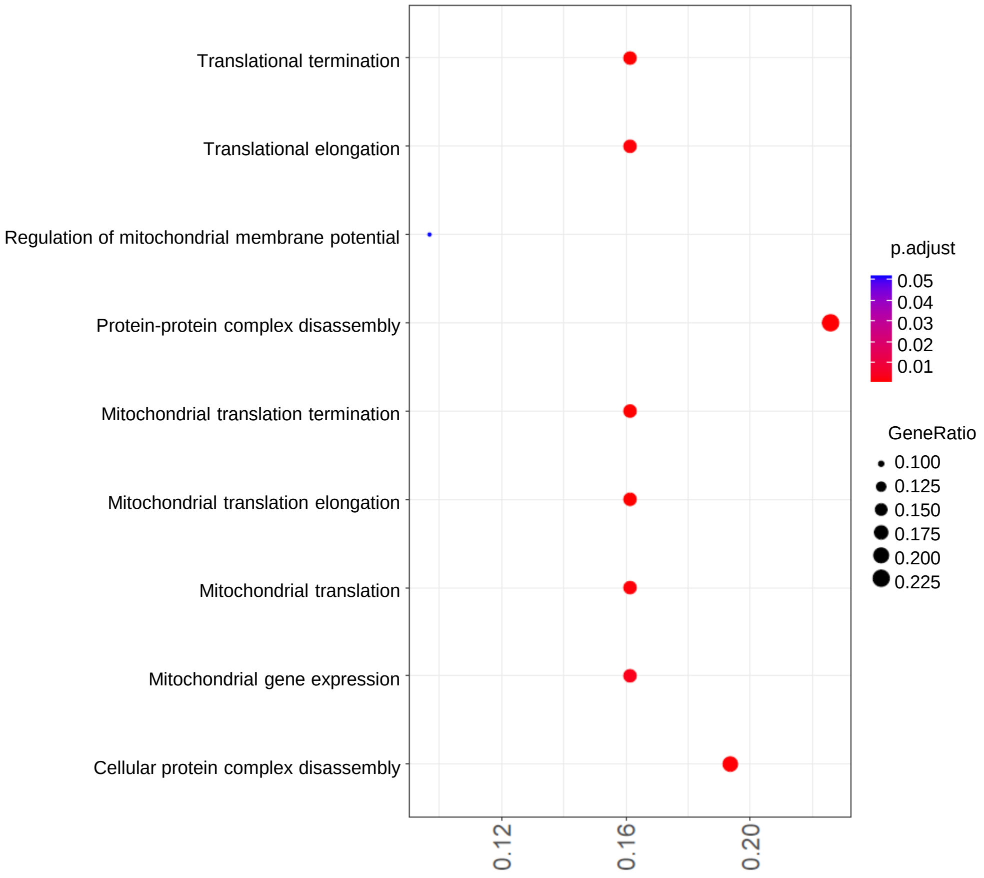

Supplementary Figure 6. Gene Ontology (GO) terms shared between maternal T cells from the chorioamniotic membranes from SARS-CoV-2 (+) women and peripheral T-cell signatures from a reference report (Meckiff et al., 2020). Over-representation analysis reporting enriched GO terms based on the common differentially expressed genes (DEGs; $q < 0.1$) between maternal T cells from the chorioamniotic membranes from SARS-CoV-2 (+) women ($n = 9$) and T cells from the reference report. Analyses were performed using one-sided Fisher's exact tests.

a

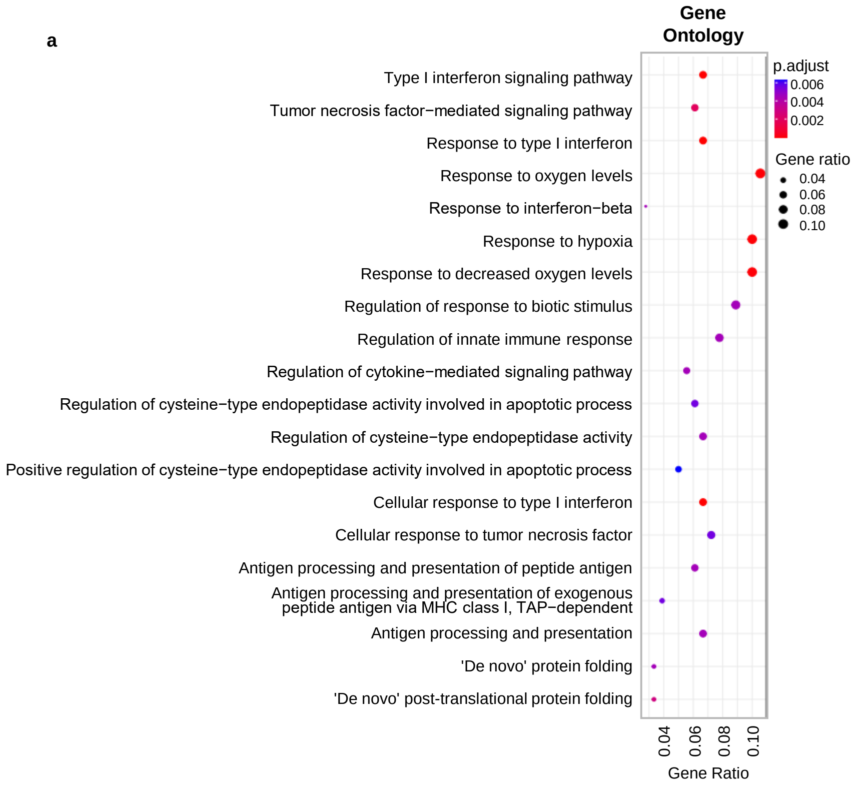

b

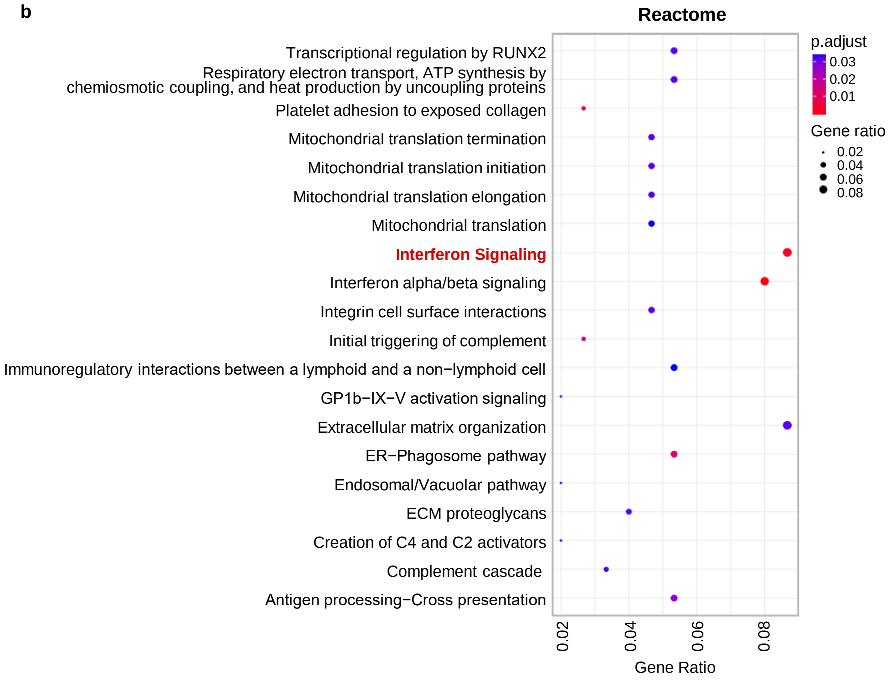

Supplementary Figure 7. Gene Ontology (GO) and Reactome pathways of combined differentially expressed genes (DEGs) in the chorioamniotic membranes (CAM) and placental villi and basal plate (PVBP) associated with SARS-CoV-2 infection. ClusterProfiler dot plots showing significant SARS-CoV-2-associated pathways ($q < 0.10$) using over-representation analyses based on one-sided Fisher's exact tests on DEGs from the CAM and PVBP [n = 10 control, 9 SARS-CoV-2 (+)] based on the **(a)** GO and **(b)** Reactome databases.

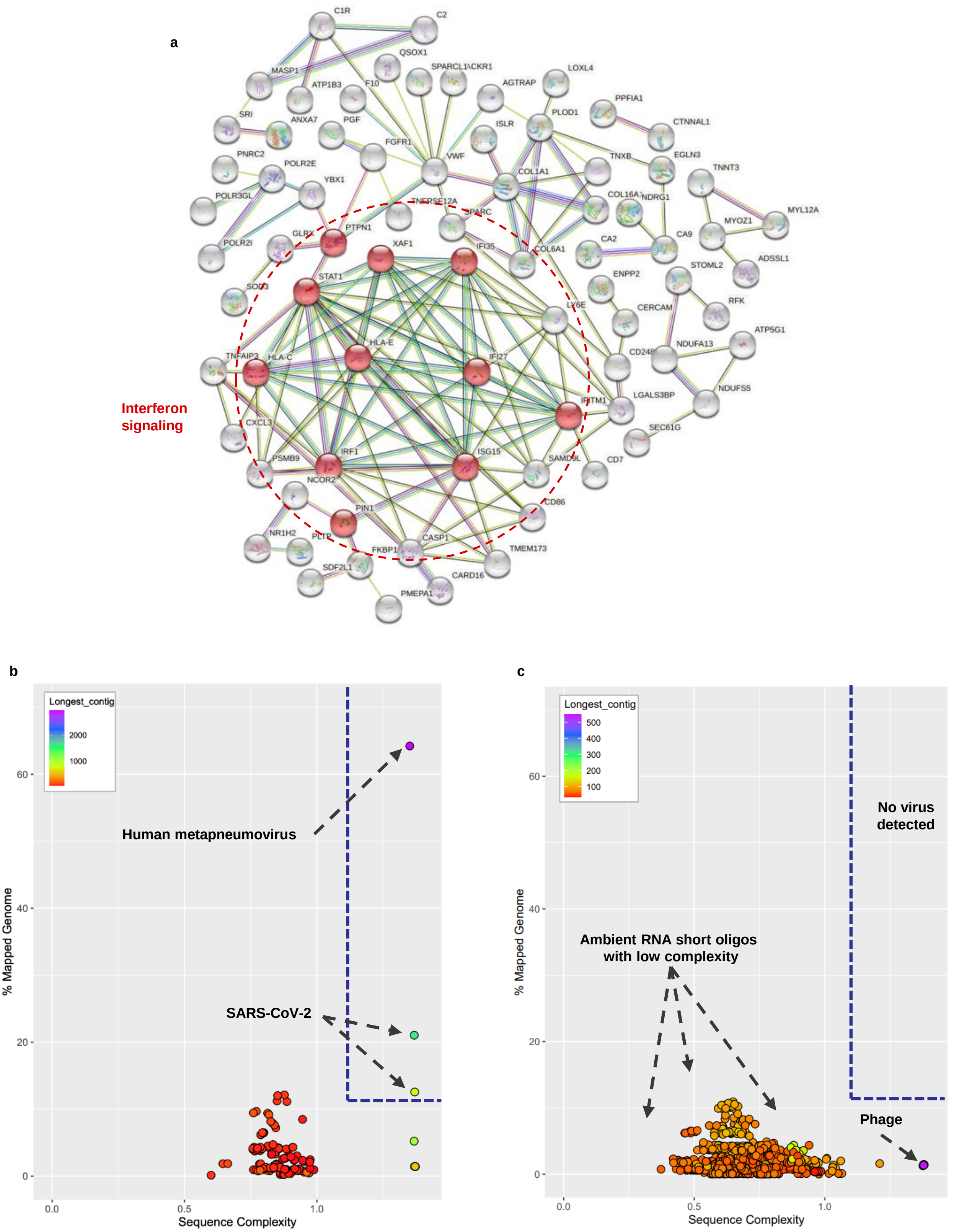

Supplementary Figure 8. STRING enrichment analysis and viral detection in the placental tissues from pregnant women with SARS-CoV-2 infection. (a) Search Tool for the Retrieval of Interacting Genes/Proteins (STRING) analysis was used to show interactions of differentially expressed genes (DEGs) associated with Interferon Signaling pathway derived from the Reactome database. DEGs were obtained from the single-cell RNA-seq data from the chorioamniotic membranes (CAM) and placental villi and basal plate (PVBP) of SARS-CoV-2 (+) (n = 9) or control (n = 10) women. Viral-Track scatter plots showing the presence of viral sequences in single-cell RNA-seq reads from **(b)** bronchoalveolar lavage of SARS-CoV-2 (+) patients derived from previously reported data (Bost et al., 2020) and **(c)** single-cell RNA-seq data from the CAM and PVBP of SARS-CoV-2 (+) and control women. Blue dashed lines represent thresholds for viral detection, with the virus name indicated by arrows.

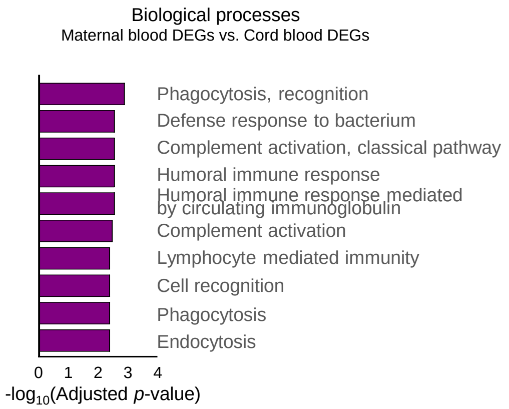

Supplementary Figure 9. Biological processes enriched among genes exhibiting a differential response to SARS-CoV-2 infection between maternal and cord blood. Bar plots showing the top 10 over-represented biological processes from Gene Ontology (GO) database based on the DEGs from the maternal blood compared to cord blood in response to SARS-CoV-2 infection with an adjusted *p*-value < 0.05. *P*-value was computed based on one-sided hypergeometric distribution and adjusted by false discovery rate.

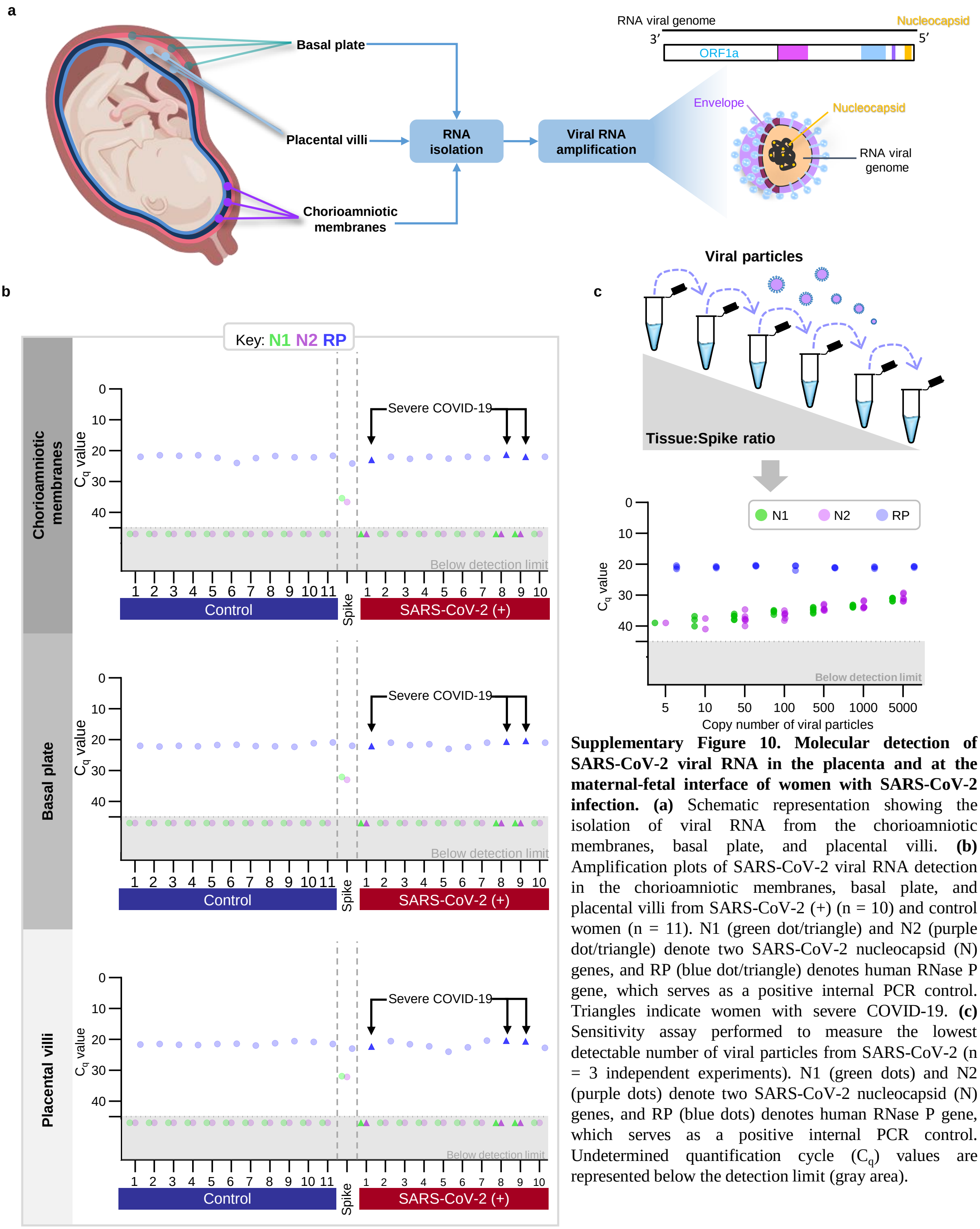

Supplementary Figure 10. Molecular detection of SARS-CoV-2 viral RNA in the placenta and at the maternal-fetal interface of women with SARS-CoV-2 infection. (a) Schematic representation showing the isolation of viral RNA from the chorioamniotic membranes, basal plate, and placental villi. **(b)** Amplification plots of SARS-CoV-2 viral RNA detection in the chorioamniotic membranes, basal plate, and placental villi from SARS-CoV-2 (+) (n = 10) and control women (n = 11). N1 (green dot/triangle) and N2 (purple dot/triangle) denote two SARS-CoV-2 nucleocapsid (N) genes, and RP (blue dot/triangle) denotes human RNase P gene, which serves as a positive internal PCR control. Triangles indicate women with severe COVID-19. **(c)** Sensitivity assay performed to measure the lowest detectable number of viral particles from SARS-CoV-2 (n = 3 independent experiments). N1 (green dots) and N2 (purple dots) denote two SARS-CoV-2 nucleocapsid (N) genes, and RP (blue dots) denotes human RNase P gene, which serves as a positive internal PCR control. Undetermined quantification cycle (C_q) values are represented below the detection limit (gray area).

Supplementary Table 1. Clinical and demographic characteristics of control women and SARS-CoV-2 (+) patients. Differences between groups were evaluated by two-sided Mann-Whitney U-tests and two-sided Fisher's exact test. P values < 0.05 were used to denote a significant result.

	Controls (n = 11)	SARS-CoV-2 (+) (n = 12)	p-value
Maternal age (years; median [IQR])[a]	29 (27-31.5)	26.5 (21-30.3)	0.3
Body mass index (kg/m^2; median [IQR])[a]	34.2 (31.8-36)	30.9 (29.1-39.4)	0.2
Primiparity[b]	18.2% (2/11)	16.7% (2/12)	1.0
Race/ethnicity[b]			1.0
African-American	100% (11/11)	91.7% (11/12)	
White	0% (0/11)	8.3% (1/12)	
Symptomatic for COVID-19	-	33.3% (4/12)	N/A
Gestational age at delivery (weeks; median [IQR])[a]	38.4 (38-39.4)	38.6 (38.4-39)[c]	0.8
Labor[b]	72.7% (8/11)	72.7% (8/11)[c]	1.0
Cesarean section[b]	45.5% (5/11)	36.4% (4/11)[c]	1.0
Preeclampsia[b]	9.1% (1/11)	9.1% (1/11)[c]	
Birthweight (grams; median [IQR])[a]	3060 (2478-3398)	3210 (2688-3260)[c]	0.9
Apgar score at 1 min (median [IQR])[a]	8 (8-8)	8 (8-8)[c]	0.1
Apgar score at 5 min (median [IQR])[a]	9 (9-9)	9 (9-9)[c]	1.0
Acute maternal inflammatory response[b]			
Stage 1 (Early acute subchorionitis or chorionitis)	18.2% (2/11)	10% (1/10)[d]	1.0
Acute fetal inflammatory response[b]			
Stage 1 (Chorionic vasculitis or umbilical phlebitis)	9.1% (1/11)	20% (2/10)[d]	0.6
Chronic maternal inflammatory response[b]			
Chronic villitis of unknown etiology	18.2% (2/11)	0% (0/10)[d]	0.5
Chronic chorioamnionitis	9.1% (1/11)	10% (1/10)[d]	1.0
Chronic fetal inflammatory response[b]			
Eosinophilic T-cell vasculitis	0% (0/11)	10% (1/10)[d]	0.5
Other placental lesions[b]			
Maternal vascular malperfusion	18.2% (2/11)	0% (0/10)[d]	0.5
Perivillous fibrin deposition	18.2% (2/11)	20% (2/10)[d]	1.0
Intervillous thrombus	9.1% (1/11)	20% (2/10)[d]	0.6
Intraplacental thrombus	9.1% (1/11)	10% (1/10)[d]	1.0

Data are given as median (interquartile range, IQR) and percentage (n/N). N/A: not applicable
[a]Mann-Whitney U test, [b]Fisher's exact test, [c]one missing datum, [d]two missing data.

Supplementary Table 2. Log_2 fold change (FC) in maternal and neonatal cytokine responses to SARS-CoV-2. For plots shown in Figure 1 and Supplementary Figures 1 and 2, undetected values were replaced with 99% of lowest detected values. Log2 fold changes and their standard errors were obtained from linear mixed models. Two-sided likelihood ratio tests were used to test the significance of each cytokine's response to SARS-CoV-2. The nominal p-values were adjusted for multiple comparisons by calculating the false discovery rate adjusted p-values (q-values).

Sample	Cytokine	log_2FC	log_2FCSE	p	q
Maternal blood	IL-8	2.55	0.59	<0.001	0.01
	IL-15	0.57	0.16	0.002	0.02
	IL-10	1.18	0.53	0.04	0.24
	IL-2	1.82	1.03	0.09	0.39
	IL-13	-1.00	0.60	0.11	0.39
	IL-7	1.03	0.63	0.12	0.39
	TNF-α	0.85	0.59	0.16	0.45
	GM-CSF	1.31	0.98	0.19	0.45
	IL-12p70	-1.46	1.16	0.22	0.45
	IL-17A	0.67	0.56	0.24	0.45
	IL-6	1.22	1.03	0.25	0.45
	IL-1β	2.16	2.31	0.36	0.59
	IL-16	0.51	0.70	0.47	0.68
	TNF-β	0.61	0.84	0.48	0.68
	IL-1α	-0.41	0.76	0.59	0.79
	IFN-ɣ	0.54	1.37	0.69	0.84
	IL-12/IL-23p40	-0.15	0.41	0.72	0.84
	IL-4	-0.44	1.51	0.77	0.84
	IL-5	-0.16	0.64	0.81	0.84
	VEGF	-0.08	0.38	0.84	0.84
Cord blood	IL-8	1.02	0.43	0.03	0.57
	IL-5	-1.31	0.90	0.16	0.82
	TNF-α	0.58	0.41	0.17	0.82
	IL-6	-1.28	0.93	0.18	0.82
	GM-CSF	-0.66	0.52	0.21	0.82
	IL-10	0.72	0.61	0.25	0.82
	VEGF	0.67	0.88	0.45	0.98
	IL-17A	0.50	0.72	0.49	0.98
	IL-12/IL-23p40	0.43	0.66	0.52	0.98
	IL-2	0.59	1.21	0.63	0.98
	IL-13	0.31	0.75	0.68	0.98
	IL-15	-0.07	0.18	0.68	0.98
	IFN-γ	-0.10	0.29	0.73	0.98
	IL-1 α	0.74	2.40	0.76	0.98
	IL-1β	-0.30	2.53	0.91	0.98
	TNF-β	-0.09	0.85	0.92	0.98
	IL-7	-0.03	0.39	0.93	0.98
	IL-12p70	0.09	1.20	0.94	0.98
	IL-4	-0.08	1.29	0.95	0.98
	IL-16	-0.02	0.69	0.98	0.98

Supplementary Table 3. Summary of slides used for immunohistological detection of SARS-CoV-2 spike and nucleocapsid proteins. Number of slides used for immunohistochemistry staining at each placental location (placental villi, basal plate, and chorioamniotic membranes) from SARS-CoV-2 (+) and control women. Internal controls used include spiked or non-spiked tissues from healthy pregnant women.

**Group**	**Individual**	**Number of slides processed for immunological detection of SARS-CoV-2 proteins**			
		Placenta villi	Basal plate	Chorioamniotic membranes	Total
Internal controls	Non-spiked tissue from healthy pregnant woman	2	2	2	6
	Spiked tissue from healthy pregnant woman	2	2	2	6
Control	#1	14	10	6	30
	#2	14	10	6	30
	#3	14	10	6	30
	#4	14	10	6	30
	#5	14	10	6	30
SARS-CoV-2 (+) women	#1 (severe COVID-19 disease)	14	10	6	30
	#2	14	10	6	30
	#3	14	10	6	30
	#4	14	10	6	30
	#5	14	10	6	30
	#6	14	10	6	30
	#7	14	10	6	30
	#8 (severe COVID-19 disease)	14	10	6	30
	#9	14	10	6	30
	#10	14	10	6	30
Total number of slides		214	154	94	462

Supplementary Table 4. Bacterial load in the placentas of women with SARS-CoV-2 infection. Comparison of bacterial load in the chorioamniotic membranes, placental amnion-chorion, and placental villous tree from SARS-CoV-2 (+) women who delivered by cesarean section or vaginally (n = 7) and from control women who delivered by cesarean section or vaginally (n = 8). The table shows no difference in the likelihood of having a bacterial load exceeding that of technical controls for background DNA contamination (i.e., blank DNA extraction kits).

	**Control**	**SARS-CoV-2 (+)**	**Proportion z-test**
**Cesarean delivery**			
Chorioamniotic membranes	1/3 (33%)	0/2 (0%)	z = 0.907, p = 0.545
Placental amnion-chorion	0/3 (0%)	1/2 (50%)	z = -1.369, p = 0.513
Placental villous tree	0/3 (0%)	2/2 (100%)	z = -2.236, p = 0.151
**Vaginal delivery**			
Chorioamniotic membranes	5/5 (100%)	5/5 (100%)	z = 0.000, p = 1.000
Placental amnion-chorion	5/5 (100%)	5/5 (100%)	z = 0.000, p = 1.000
Placental villous tree	4/5 (80%)	5/5 (100%)	z = -1.054, p = 0.545

[a]Two-tailed, with Benjamini-Hochberg corrections applied

Supplementary Table 5. Antibodies used for immunophenotyping.

Antibody	Fluorophore	Clone	Company	Dilution factor
Isotype	PE-Cy7	RTK4530	BioLegend	20
Isotype	BV711	X40	BD Biosciences	20
Isotype	PE-Cy5	Clone MOPC-21	BD Biosciences	5
Isotype	APC	Clone MOPC-21	BD Biosciences	5
Isotype	PE-CF594	Clone X40	BD Biosciences	20
Isotype	Alexa488	Clone 27-35	BD Biosciences	20
CD3	BUV737	UCHT1 (also known as UCHT-1, UCHT 1)	BD Biosciences	50
CD4	APC-H7	RPA-T4	BD Biosciences	20
CD8α	BUV395	RPA-T8	BD Biosciences	50
CD45RA	Alexa 700	HI100	BD Biosciences	20
CD196 (CCR6)	Alexa 488	G034E3	BioLegend	20
CD197 (CCR7)	PE-Cy7	3D12	BD Biosciences	20
CD45	V450	HI30	BD Biosciences	20
CD183 (CXCR3)	APC	1C6/CXCR3 (also known as 1C6, LS177-1C6)	BD Biosciences	5
CD3	APC-H7	SK7	BD Biosciences	20
CD19	PE-Cy5	HIB19	BD Biosciences	10
CD14	BUV395	MφP9	BD Biosciences	20
CD45	Alexa700	HI30	BD Biosciences	20
CD4	BUV737	SK3	BD Biosciences	20
CD15	BV650	HI98	BD Biosciences	33
CD8	BV786	RPA-T8	BD Biosciences	50
CD56	BV711	NCAM16.2	BD Biosciences	20
CCR5 (CD195)	BV711	2D7/CCR5	BD Biosciences	20
CD181 (CXCR1)	PE-Cy5	5A12	BD Biosciences	5
CD182 (CXCR2)	APC	6C6	BD Biosciences	5
CD103	PE-Cy7	Ber-ACT8	BioLegend	20
Granzyme B	PE-CF594	GB11	BD Biosciences	20
Perforin	Alexa488	δG9	BD Biosciences	20
CD62L	BV650	DREG-56	BD Biosciences	20
CD45RO	PE-Cy5	UCHL1	BD Biosciences	10
CD300a	PE	E59.126	Beckman Coulter	5
HLA-DR	PE-CF594	G46-6	BD Biosciences	20
CD2	BV421	RPA-2.10	BD Biosciences	20
CD19	Alexa488	HIB19	BD Biosciences	20
CD25	PE-Cy7	M-A251	BD Biosciences	20
CD29	BV510	4-Mar	BD Biosciences	20
CD30	APC	BerH8	BD Biosciences	20
CD54	BV711	HA58	BD Biosciences	20
CD57	BV605	QA17A04	BioLegend	20
CD122 (IL-2Rβ)	PE	Clone TU27	BioLegend	20
CD14	BV650	M5E2	BD Biosciences	20
CD15	BV605	W6D3	BD Biosciences	50
CD16	PerCP-Cy5.5	Clone 3G8	BD Biosciences	20
CD62L	PE-CF594	DREG-56	BD Biosciences	20
CD279	FITC	MIH4	BD Biosciences	20
CD64	APC-H7	10.1	BD Biosciences	10
CX3CR1	PE-Cy7	2A9-1	BioLegend	20
