# Supplementary material for: Maternal-fetal immune responses in pregnant women infected with SARS-CoV-2
Source: Nat Commun. 2022 Jan 18;13:320. doi: 10.1038/s41467-021-27745-z (PMC8766450; doi:10.1038/s41467-021-27745-z)
Supplement: Supplementary file 3 — Description of Additional Supplementary Files [file 41467_2021_27745_MOESM3_ESM.pdf]

Description of Additional Supplementary Files

File Name: Supplementary Data 1
Description: Differentially expressed genes (DEGs) in the CAM and PVBP from women with SARS-CoV-2 infection

File Name: Supplementary Data 2
Description: Differentially expressed genes (DEGs) in the maternal blood from women with SARS-CoV-2 infection

File Name: Supplementary Data 3
Description: KEGG pathways enriched in the differentially expressed genes (DEGs) in the maternal blood from women with SARS-CoV-2 infection

File Name: Supplementary Data 4
Description: Differentially expressed genes (DEGs) in the cord blood from women with SARS-CoV-2 infection

File Name: Supplementary Data 5
Description: KEGG pathways enriched in the differentially expressed genes (DEGs) in the cord blood from women with SARS-CoV-2 infection.

File Name: Supplementary Data 6
Description: List of differentially expressed genes (DEGs) from the interaction analysis between maternal and cord blood in response to SARS-CoV-2 infection

File Name: Supplementary Data 7
Description: List of genes utilized to assign the cell type identities in CAM and PVBP
