# Supplementary material for: Maternal-fetal immune responses in pregnant women infected with SARS-CoV-2
Source: Nat Commun. 2022 Jan 18;13:320. doi: 10.1038/s41467-021-27745-z (PMC8766450; doi:10.1038/s41467-021-27745-z)
Supplement: Supplementary file 11 — Reporting Summary [file 41467_2021_27745_MOESM11_ESM.pdf]

nature research

Corresponding author(s): Nardhy Gomez-Lopez

Last updated by author(s): 11/17/2021

Reporting Summary

Nature Research wishes to improve the reproducibility of the work that we publish. This form provides structure for consistency and transparency in reporting. For further information on Nature Research policies, see our Editorial Policies and the Editorial Policy Checklist.

Statistics

For all statistical analyses, confirm that the following items are present in the figure legend, table legend, main text, or Methods section.

n/a	Confirmed	
☐	☒	The exact sample size (n) for each experimental group/condition, given as a discrete number and unit of measurement
☐	☒	A statement on whether measurements were taken from distinct samples or whether the same sample was measured repeatedly
☐	☒	The statistical test(s) used AND whether they are one- or two-sided<br>*Only common tests should be described solely by name; describe more complex techniques in the Methods section.*
☒	☐	A description of all covariates tested
☐	☒	A description of any assumptions or corrections, such as tests of normality and adjustment for multiple comparisons
☐	☒	A full description of the statistical parameters including central tendency (e.g. means) or other basic estimates (e.g. regression coefficient) AND variation (e.g. standard deviation) or associated estimates of uncertainty (e.g. confidence intervals)
☐	☒	For null hypothesis testing, the test statistic (e.g. F, t, r) with confidence intervals, effect sizes, degrees of freedom and P value noted<br>*Give P values as exact values whenever suitable.*
☒	☐	For Bayesian analysis, information on the choice of priors and Markov chain Monte Carlo settings
☐	☒	For hierarchical and complex designs, identification of the appropriate level for tests and full reporting of outcomes
☐	☒	Estimates of effect sizes (e.g. Cohen's d, Pearson's r), indicating how they were calculated

Our web collection on statistics for biologists contains articles on many of the points above.

Software and code

Policy information about availability of computer code

Data collection: FACSDiva 6.0 from BD Biosciences was used to acquire flow cytometry data.

Data analysis: Genotyping: genotype information was converted to vcf format using “iaap-cli gencall” and “gtc_to_vcf.py” from Illumina, and imputation to 37.5 M variants using the 1000 Genomes (https://www.internationalgenome.org/) haplotype references was done using the University of Michigan Imputation Server (https://imputationserver.sph.umich.edu/). The maternal/fetal relationship of the genotyped samples was ascertained using plink2 KING-robust kinship analysis.

RNAseq analysis: Fastq files were aligned using kallisto, and bustools summarized the cell/gene transcript counts in a matrix for each sample. Each library was then processed using DIEM to eliminate debris and empty droplets. Cell Ranger version 4.0.0 (10X Genomics) was also used to align the scRNAseq reads using the STAR aligner to produce the bam files necessary for demultiplexing the individual of origin, based on the genotype information using souporcell (https://github.com/wheaton5/souporcell) and demuxlet (https://github.com/statgen/demuxlet). All count data matrices were then normalized and combined using Seurat version 3.1 (https://satijalab.org/seurat/). Different libraries were integrated and harmonized using the Harmony version 1.0 (https://portals.broadinstitute.org/harmony/articles/quickstart.html) To label the cells, the SingleR package version 1.3.8 (https://doi.org/doi:10.18129/B9.bioc.SingleR) was used to assign a cell-type identity. For differential gene expression, the negative binomial model implemented in DESeq2 version 1.28.1 (https://doi.org/doi:10.18129/B9.bioc.DESeq2) was used. ggplot2 R package version 3.3.2 (https://ggplot2.tidyverse.org/) was used to visualize the comparison between data generated in this study and a prior dataset, and the genes obtained from the combined datasets were further analyzed for gene set enrichment analysis (GSEA) and over-representation analysis (ORA) with clusterProfiler version 4.0.1 (https://doi.org/doi:10.18129/B9.bioc.clusterProfiler). The R-based computational pipeline Viral-Track was used to study viruses in raw scRNAseq data (github.com/PierreBSC/Viral-Track). The viral genomes were downloaded from the Virusite database version 2020.3 that includes all published viruses, viroids, and satellites (NCBI RefSeq).

Bulk RNA-seq analysis: Transcript abundance from RNA-seq reads was quantified with Salmon (https://github.com/COMBINE-lab/Salmon). The differential expression of genes between groups was tested by fitting a negative binomial distribution model implemented in DESeq2 version

1.28.1 (https://doi.org/doi:10.18129/B9.bioc.DESeq2). The differentially expressed genes for each group comparison were used as input in iPathwayGuide (https://advaitabio.com/ipathwayguide/) to identify the significantly impacted biological processes and pathways.

MiSeq analysis: 16S rRNA gene sequences were clustered into amplicon sequence variants (ASVs) defined by 100% sequence similarity using DADA2 version 1.12 according to the online MiSeq protocol (https://benjjneb.github.io/dada2/tutorial.html). Sequences were then classified using the silva_nr_v132_train_set database. decontam version 1.6.0 was used to identify amplicon sequence variants that were potential background DNA contaminants. Heatmaps of the 16S rRNA gene profiles of samples were generated using the open-source software program Morpheus (https://software.broadinstitute.org/morpheus). Variation in the 16S rRNA gene profiles were visualized through Principal Coordinates Analyses (PCoA) using the R package vegan version 2.5-6.

Flow cytometry analysis was performed using FlowJo version 10.

Statistical analysis: Statistical analyses were performed using SPSS v19.0 (IBM, Armonk, NY, USA) or the R package.

For manuscripts utilizing custom algorithms or software that are central to the research but not yet described in published literature, software must be made available to editors and reviewers. We strongly encourage code deposition in a community repository (e.g. GitHub). See the Nature Research guidelines for submitting code & software for further information.

Data

Policy information about availability of data

All manuscripts must include a data availability statement. This statement should provide the following information, where applicable:

- Accession codes, unique identifiers, or web links for publicly available datasets
- A list of figures that have associated raw data
- A description of any restrictions on data availability

The majority of the data generated in this study are included in the manuscript or in the Supplementary Materials. The genotyping and single-cell RNAseq data reported in this study have been submitted to the NIH dbGAP repository (accession number phs001886.v3.p1). All software and R packages used herein are detailed in the Materials and Methods. Scripts detailing the single-cell analyses are also available at https://github.com/piquelab/covid19placenta. The raw MiSeq data reported in this study have been deposited in the NCBI Sequence Read Archive (Bioproject ID: PRJNA701628). The bulk RNA-seq data from the maternal and cord blood were deposited in the Gene Expression Omnibus (accession number GSE: pending).

The STRING database (https://string-db.org) was utilized to identify and visualize the enrichment of GO terms among all differentially expressed genes.

Field-specific reporting

Please select the one below that is the best fit for your research. If you are not sure, read the appropriate sections before making your selection.

☒ Life sciences ☐ Behavioural & social sciences ☐ Ecological, evolutionary & environmental sciences

For a reference copy of the document with all sections, see nature.com/documents/nr-reporting-summary-flat.pdf

Life sciences study design

All studies must disclose on these points even when the disclosure is negative.

Sample size	Sample size calculations were not performed. Given the difficulty in obtaining samples from pregnant women positive for SARS-CoV-2, we included the maximum number of samples that could be collected within a reasonable time frame. The number of samples from control (SARS-CoV-2-negative) pregnant women were matched as closely as possible to those of the cases for each experiment to ensure similar statistical power.
Data exclusions	Data were not excluded from analysis.
Replication	Given the novelty of studying pregnant women with COVID-19 and the limited patient samples available, we were unable to replicate our findings in a separate patient cohort. Yet, our findings are largely consistent with other studies and cases reports in non-pregnant and pregnant patients, which we discuss in our manuscript.
Randomization	Patient randomization was not performed, as the study was focused on SARS-CoV-2-positive pregnant women and healthy pregnant women. We report that there were no differences in clinical and demographic covariates between the two study groups.
Blinding	The study was centered on the SARS-CoV-2 status of each patient, which for safety reasons was determined prior to sample collection and experimentation. Data collection and analysis were performed using computational pipelines or objective experimental thresholds that were applied equally to all samples.

Reporting for specific materials, systems and methods

We require information from authors about some types of materials, experimental systems and methods used in many studies. Here, indicate whether each material, system or method listed is relevant to your study. If you are not sure if a list item applies to your research, read the appropriate section before selecting a response.

Materials & experimental systems

n/a	Involved in the study	
☐	☒	Antibodies
☒	☐	Eukaryotic cell lines
☒	☐	Palaeontology and archaeology
☒	☐	Animals and other organisms
☐	☒	Human research participants
☒	☐	Clinical data
☒	☐	Dual use research of concern

Methods

n/a	Involved in the study	
☒	☐	ChIP-seq
☐	☒	Flow cytometry
☒	☐	MRI-based neuroimaging

Antibodies

Antibodies used

Antibody, Name supplier, Catalog No, Clone name, Lot. No
Immunophenotyping
Isotype PE, BD Biosciences, Catalog No: 554648, Clone name: G155-178, Lot. No: 923862
Isotype PerCPCy5.5, BD Biosciences, Catalog No: 552834, Clone name: MOPC-21, Lot. No: 0079255
Isotype FITC, BD Biosciences, Catalog No: 340755, Clone name: X40, Lot. No: 9267160
Isotype A647, BioLegend, Catalog No: 557783, Clone name: MOPC-21, Lot. No: 9185417
Isotype BV421, BD Biosciences, Catalog No: 562748, Clone name: 27-35, Lot. No: 9301756
Isotype BV605, BioLegend, Catalog No: 400162, Clone name: MOPC-21, Lot. No: B310827
Isotype Alexa594, BD Biosciences, Catalog No: 562309, Clone name: R3-34, Lot. No: 0003996
Isotype PE-Cy7, BioLegend, Catalog No: 400618, Clone name: RTK4530, Lot. No: B266046
Isotype BV711, BD Biosciences, Catalog No: 563044, Clone name: X40, Lot. No: 8262906
Isotype PE-Cy5, BD Biosciences, Catalog No: 555750, Clone name: MOPC-21, Lot. No: 9066776
Isotype APC, BD Biosciences, Catalog No: 555751, Clone name: MOPC-21, Lot. No: 9038517
Isotype PE, BD Biosciences, Catalog No: 555749, Clone name: MOPC-21, Lot. No: 3046675
Isotype PE-CF594, BD Biosciences, Catalog No: 562292, Clone name: X40, Lot. No: 6083964
Isotype BV650, BD Biosciences, Catalog No: 563231, Clone name: X40, Lot. No: 0149138
Isotype BV786, BD Biosciences, Catalog No: 563847, Clone name: R3-34, Lot. No: 9345911
Isotype BV510, BD Biosciences, Catalog No: 562952, Clone name: R35-95, Lot. No: 0209308
Isotype BV711, BD Biosciences, Catalog No: 563283, Clone name: R3-34, Lot. No: 0240968
Isotype Alexa488, BD Biosciences, Catalog No: 558716, Clone name: 27-35, Lot. No: 0086827
Isotype Alexa700, BD Biosciences, Catalog No: 557882, Clone name: MOPC-21, Lot. No: 9322735
Isotype PE, BD Biosciences, Catalog No: 554685, Clone name: R3-34, Lot. No: 9141990
Isotype Alexa647, BD Biosciences, Catalog No: 400136, Clone name: MOPC-21, Lot. No: B287199
CD3 BUV737, BD Biosciences, Catalog No: 612750, Clone name: UCHT1 (also known as UCHT-1, UCHT 1), Lot. No: 9212190
CD4 APC-H7, BD Biosciences, Catalog No: 560158, Clone name: RPA-T4, Lot. No: 0029404
CD8α BUV395, BD Biosciences, Catalog No: 563795, Clone name: RPA-T8, Lot. No: 9346411
CD45RA Alexa 700, BD Biosciences, Catalog No: 560673, Clone name: HI100, Lot. No: 6182503
CD196 (CCR6) Alexa 488, BioLegend, Catalog No: 353414, Clone name: G034E3, Lot. No: B255705
CD197 (CCR7) PE-Cy7, BD Biosciences, Catalog No: 557648, Clone name: 3D12, Lot. No: 9136737
CD45V450, BD Biosciences, Catalog No: 560367, Clone name: HI30, Lot. No: 9289771
CD183 (CXCR3) APC, BD Biosciences, Catalog No: 550967, Clone name: 1C6/CXCR3 (also known as 1C6, LS177-1C6), Lot. No: 9105766
CD3 APC-H7, BD Biosciences, Catalog No: 557832, Clone name: SK7, Lot. No: 9275889
CD19 PE-Cy5, BD Biosciences, Catalog No: 555414, Clone name: HIB19, Lot. No: 9155523
CD14 BUV395, BD Biosciences, Catalog No: 563561, Clone name: MφP9, Lot. No: 0119039
CD45 Alexa700, BD Biosciences, Catalog No: 560566, Clone name: HI30, Lot. No: 0016145
CD4 BUV737, BD Biosciences, Catalog No: 612748, Clone name: SK3, Lot. No: 9276448
CD15 BV650, BD Biosciences, Catalog No: 564232, Clone name: HI98, Lot. No: 9337408
CD8 BV786, BD Biosciences, Catalog No: 563823, Clone name: RPA-T8, Lot. No: 9182913
CD56 BV711, BD Biosciences, Catalog No: 563169, Clone name: NCAM16.2, Lot. No: 0041400
MIP1α PE, BD Biosciences, Catalog No: 554730, Clone name: 11A3, Lot. No: 9165847
MIP1β PerCPCy5.5, BD Biosciences, Catalog No: 560688, Clone name: D21-1351, Lot. No: 0024543
IL-1α FITC, BD Biosciences, Catalog No: 340513, Clone name: AS5, Lot. No: 9165780
IL-1β Alexa647, BioLegend, Catalog No: 508208, Clone name: JK1B-1, Lot. No: B274564
IL-8 BV421, BD Biosciences, Catalog No: 563310, Clone name: G265-8, Lot. No: 9165979
TNFα BV605, BioLegend, Catalog No: 502936, Clone name: MAb11, Lot. No: B298444
IL-6 Alexa594, BD Biosciences, Catalog No: 563543, Clone name: MQ2-13A5, Lot. No: 0170548
CX3CR1PE-Cy7, BioLegend, Catalog No: 341612, Clone name: 2A9-1, Lot. No: B298605
CCR5 (CD195) BV711, BD Biosciences, Catalog No: 563395, Clone name: 2D7/CCR5, Lot. No: 1117612
CD181 (CXCR1) PE-Cy5, BD Biosciences, Catalog No: 551081, Clone name: 5A12, Lot. No: 0282711
CD182 (CXCR2)APC, BD Biosciences, Catalog No: 551127, Clone name: 6C6, Lot. No: 0314355
IL-1β FITC, BD Biosciences, Catalog No: 340515, Clone name: AS10, Lot. No: 9311369
IL-1RA PE, BD Biosciences, Catalog No: 340525, Clone name: AS17, Lot. No: 0258978

CD103 PE-Cy7, BioLegend, Catalog No: 350212, Clone name: Ber-ACT8, Lot. No: B262937
Granzyme B PE-CF594, BD Biosciences, Catalog No: 562462, Clone name: GB11, Lot. No: 1005787
IFNɣ BV650, BD Biosciences, Catalog No: 563416, Clone name: 4S.B3, Lot. No: 0246087
IL-10 BV786, BD Biosciences, Catalog No: 564049, Clone name: JES3-9D7, Lot. No: 9009679
IL-2 BV510, BD Biosciences, Catalog No: 564167, Clone name: MQ1-17H12, Lot. No: 9072658
IL-4 BV711, BD Biosciences, Catalog No: 564112, Clone name: MP4-25D2, Lot. No: 9095675
Perforin Alexa488, BD Biosciences, Catalog No: 563764, Clone name: δG9, Lot. No: 9322656
IL-17A Alexa700, BD Biosciences, Catalog No: 560613, Clone name: N49-653, Lot. No: 0156497
IL-5 PE, BD Biosciences, Catalog No: 554395, Clone name: TRFK5, Lot. No: 8130811
IL-9 PerCP-Cy5.5, BD Biosciences, Catalog No: 561461, Clone name: MH9A3, Lot. No: 9116799
T-bet Alexa647, BD Biosciences, Catalog No: 561264, Clone name: 4B10, Lot. No: 0279857
CD62L BV650, BD Biosciences, Catalog No: 563808, Clone name: DREG-56, Lot. No: 9207222
CD45RO PE-Cy5, BD Biosciences, Catalog No: 555494, Clone name: UCHL1, Lot. No: 9108529
CD300a PE, Beckman Coulter, Catalog No: A22328, Clone name: E59.126, Lot. No: 200029
HLA-DR PE-CF594, BD Biosciences, Catalog No: 562304, Clone name: G46-6, Lot. No: 9155784
CD2 BV421, BD Biosciences, Catalog No: 562639, Clone name: RPA-2.10, Lot. No: 8254825
CD19 Alexa488, BD Biosciences, Catalog No: 557697, Clone name: HIB19, Lot. No: 9080789
CD25 PE-Cy7, BD Biosciences, Catalog No: 557741, Clone name: M-A251, Lot. No: 1068706
CD29 BV510, BD Biosciences, Catalog No: 747747, Clone name: MAR4, Lot. No: 9346335
CD30 APC, BD Biosciences, Catalog No: 563500, Clone name: BerH8, Lot. No: 9199297
CD54 BV711, BD Biosciences, Catalog No: 564078, Clone name: HA58, Lot. No: 9171772
CD57 BV605, BioLegend, Catalog No: 393304, Clone name: QA17A04, Lot. No: B299310
CD122 (IL-2Rβ) PE, BioLegend, Catalog No: 339006, Clone name: Clone TU27, Lot. No: B300806
CD10 BUV395, BD Biosciences, Catalog No: 563871, Clone name: HI10a, Lot. No: 8262696
CD13 PE-Cy7, BD Biosciences, Catalog No: 338425, Clone name: L138, Lot. No: 0059888
CD14 BV650, BD Biosciences, Catalog No: 563419, Clone name: M5E2, Lot. No: 9093937
CD15 BV605, BD Biosciences, Catalog No: 562980, Clone name: W6D3, Lot. No: 9266360
CD16 PerCP-Cy5.5, BD Biosciences, Catalog No: 560717, Clone name: 3G8, Lot. No: 9325110
CD33 BV711, BD Biosciences, Catalog No: 563171, Clone name: WM53, Lot. No: 9336117
CD62L PE-CF594, BD Biosciences, Catalog No: 562301, Clone name: DREG-56, Lot. No: 9196191
CD123 BV786, BD Biosciences, Catalog No: 564196, Clone name: 7G3, Lot. No: 0233594
CD185 Alexa700/APC-Cy5.5, BioLegend, Catalog No: 356916, Clone name: J252D4, Lot. No: B254830
CD203c BV421, BD Biosciences, Catalog No: 563296, Clone name: NP4D6, Lot. No: 9316914
CD279 FITC, BD Biosciences, Catalog No: 557860, Clone name: MIH4, Lot. No: 9352010
CD64 APC-H7, BD Biosciences, Catalog No: 561190, Clone name: 10.1, Lot. No: 0134465
CD303 APC/Alexa647, BioLegend, Catalog No: 354206, Clone name: 201A, Lot. No: B287410
CD83 PE-Cy5, BD Biosciences, Catalog No: 551058, Clone name: HB15e, Lot. No: 8330949
CD172b PE, BD Biosciences, Catalog No: 552602, Clone name: B4B6, Lot. No: 9119970
Immunohistochemistry
Universal mouse isotype, Agilent, Catalog No: IS75061-2, Clone name: n/a, Lot. No: 10146824
Universal rabbit isotype, Agilent, Catalog No: IS60061-2, Clone name: n/a, Lot. No: 11301627
SARS-CoV-2 (COVID-19) spike, GeneTex, Catalog No: GTX632604, Clone name: n/a, Lot. No: 44168
SARS-CoV-2 (COVID-19) nucleocapsid, GeneTex, Catalog No: GTX135357, Clone name: n/a, Lot. No: 43957

Validation

Immunophenotyping (Antibody, Species, Application, Sample used for validation, Dilution factor used for validation):
Isotype PE, Human, Flow cytometry, PBMCs stimulated with LPS, dilution factor tested in house
Isotype PerCPCy5.5, Human, Flow cytometry, PBMCs stimulated with human IFN-γ and LPS, dilution factor recommended by manufacturer
Isotype FITC, Human, Flow cytometry, PBMCs stimulated with LPS, dilution factor tested in house
Isotype A647, Human, Flow cytometry, PBMCs stimulated with LPS, dilution factor recommended by manufacturer
Isotype V421, Human, Flow cytometry, PBMCs stimulated with LPS, dilution factor tested in house
Isotype BV605, Human, Flow cytometry, PBMCs stimulated with PMA plus ionomycin, recommended by manufacturer
Isotype Alexa594, Human, Flow cytometry, PBMCs stimulated with LPS, dilution factor tested in house
Isotype PE-Cy7, Human, Flow cytometry, Peripheral blood, dilution factor recommended by manufacturer
Isotype BV711, Human, Flow cytometry, Peripheral blood, dilution factor recommended by manufacturer
Isotype PE-Cy5, Human, Flow cytometry, Peripheral blood, dilution factor recommended by manufacturer
Isotype APC, Human, Flow cytometry, Peripheral blood, dilution factor recommended by manufacturer
Isotype PE, Human, Flow cytometry, PBMCs stimulated with LPS, dilution factor tested in house
Isotype PE-CF594, Human, Flow cytometry, PBMCs, dilution factor recommended by manufacturer
Isotype BV650, Human, Flow cytometry, PBMCs stimulated with PMA plus ionomycin, dilution factor tested in house
Isotype BV786, Human, Flow cytometry, PBMCs stimulated with LPS, dilution factor recommended by manufacturer
Isotype BV510, Human, Flow cytometry, PBMCs stimulated with PMA plus ionomycin, dilution factor recommended by manufacturer
Isotype BV711, Human, Flow cytometry, PBMCs stimulated with PMA plus ionomycin, dilution factor recommended by manufacturer
Isotype Alexa488, Human, Flow cytometry, PBMCs, dilution factor recommended by manufacturer
Isotype Alexa700, Human, Flow cytometry, PBMCs stimulated with PMA plus ionomycin, dilution factor recommended by manufacturer
Isotype PE, Human, Flow cytometry, PBMCs stimulated with immobilized anti-human CD3 + soluble anti-human CD28 + IL-2 + IL-4 and restimulated with PMAplus ionomycin, dilution factor tested in house
Isotype Alexa647, Human, Flow cytometry, PBMCs stimulated with PMA plus ionomycin, dilution factor tested in house

CD3 BUV737, Human, Flow cytometry, Peripheral blood, dilution factor tested in house
CD4 APC-H7, Human, Flow cytometry, Peripheral blood, dilution factor recommended by manufacturer
CD8α BUV395, Human, Flow cytometry, Peripheral blood, dilution factor tested in house
CD45RA Alexa 700, Human, Flow cytometry, Peripheral blood, dilution factor recommended by manufacturer
CD196 (CCR6) Alexa 488, Human, Flow cytometry, Peripheral blood, dilution factor recommended by manufacturer
CD197 (CCR7) PE-Cy7, Human, Flow cytometry, Peripheral blood, dilution factor recommended by manufacturer
CD45V450, Human, Flow cytometry, Peripheral blood, dilution factor recommended by manufacturer
CD183 (CXCR3) APC, Human, Flow cytometry, Peripheral blood, dilution factor recommended by manufacturer
CD3 APC-H7, Human, Flow cytometry, Peripheral blood, dilution factor recommended by manufacturer
CD19 PE-Cy5, Human, Flow cytometry, Peripheral blood, dilution factor recommended by manufacturer
CD14 BUV395, Human, Flow cytometry, Peripheral blood, dilution factor recommended by manufacturer
CD45 Alexa700, Human, Flow cytometry, Peripheral blood, dilution factor recommended by manufacturer
CD4 BUV737, Human, Flow cytometry, Peripheral blood, dilution factor recommended by manufacturer
CD15 BV650, Human, Flow cytometry, Peripheral blood, dilution factor tested in house
CD8 BV786, Human, Flow cytometry, Peripheral blood, dilution factor tested in house
CD56 BV711, Human, Flow cytometry, Peripheral blood, dilution factor recommended by manufacturer
MIP1α PE, Human, Flow cytometry, PBMCs stimulated with LPS, dilution factor recommended by manufacturer
MIP1β PerCPCy5.5, Human, Flow cytometry, PBMCs stimulated with human IFN-γ and LPS, dilution factor recommended by manufacturer
IL-1α FITC, Human, Flow cytometry, PBMCs stimulated with LPS, dilution factor recommended by manufacturer
IL-1β Alexa647, Human, Flow cytometry, PBMCs stimulated with LPS, dilution factor recommended by manufacturer
IL-8 BV421, Human, Flow cytometry, PBMCs stimulated with LPS, dilution factor recommended by manufacturer
TNFα BV605, Human, Flow cytometry, PBMCs stimulated with PMA plus ionomycin, dilution factor recommended by manufacturer
IL-6 Alexa594, Human, Flow cytometry, PBMCs stimulated with LPS, dilution factor recommended by manufacturer
CX3CR1 PE-Cy7, Human, Flow cytometry, Peripheral blood, dilution factor recommended by manufacturer
CCR5 (CD195) BV711, Human, Flow cytometry, Peripheral blood, dilution factor recommended by manufacturer
CD181 (CXCR1) PE-Cy5, Human, Flow cytometry, Peripheral blood, dilution factor recommended by manufacturer
CD182 (CXCR2)APC, Human, Flow cytometry, Peripheral blood, dilution factor recommended by manufacturer
IL-1β FITC, Human, Flow cytometry, PBMCs stimulated with LPS, dilution factor recommended by manufacturer
IL-1RA PE, Human, Flow cytometry, PBMCs stimulated with LPS, dilution factor recommended by manufacturer
CD103 PE-Cy7, Human, Flow cytometry, PBMCs, dilution factor recommended by manufacturer
Granzyme B PE-CF594, Human, Flow cytometry, PBMCs, dilution factor recommended by manufacturer
IFNɣ BV650, Human, Flow cytometry, PBMCs stimulated with PMA plus ionomycin, dilution factor recommended by manufacturer
IL-10 BV786, Human, Flow cytometry, PBMCs stimulated with LPS, dilution factor recommended by manufacturer
IL-2 BV510, Human, Flow cytometry, PBMCs stimulated with PMA plus ionomycin, dilution factor recommended by manufacturer
IL-4 BV711, Human, Flow cytometry, PBMCs stimulated with PMA plus ionomycin, dilution factor recommended by manufacturer
Perforin Alexa488, Human, Flow cytometry, PBMCs, dilution factor recommended by manufacturer
IL-17A Alexa700, Human, Flow cytometry, PBMCs stimulated with PMA plus ionomycin, dilution factor tested in house
IL-5 PE, Human, Flow cytometry, PBMCs stimulated with immobilized anti-human CD3 + soluble anti-human CD28 + IL-2 + IL-4 and restimulated with PMA plus ionomycin, dilution factor tested in house
IL-9 PerCP-Cy5.5, Human, Flow cytometry, PBMCs stimulated with immobilized anti-human CD3 + soluble anti-human CD28 + IL-2 + IL-4 and restimulated with PMA plus ionomycin, dilution factor tested in house
T-bet Alexa647, Human, Flow cytometry, PBMCs stimulated with PMA plus ionomycin, dilution factor tested in house
CD62L BV650, Human, Flow cytometry, Peripheral blood, dilution factor recommended by manufacturer
CD45RO PE-Cy5, Human, Flow cytometry, Peripheral blood, dilution factor recommended by manufacturer
CD300a PE, Human, Flow cytometry, Peripheral blood, dilution factor recommended by manufacturer
HLA-DR PE-CF594, Human, Flow cytometry, Peripheral blood, dilution factor recommended by manufacturer
CD2 BV421, Human, Flow cytometry, Peripheral blood, dilution factor recommended by manufacturer
CD19 Alexa488, Human, Flow cytometry, Peripheral blood, dilution factor recommended by manufacturer
CD25 PE-Cy7, Human, Flow cytometry, PBMCs stimulated with PHA, dilution factor recommended by manufacturer
CD29 BV510, Human, Flow cytometry, PBMCs, dilution factor recommended by manufacturer
CD30 APC, Human, Flow cytometry, PBMCs stimulated with PHA, dilution factor recommended by manufacturer
CD54 BV711, Human, Flow cytometry, Peripheral blood, dilution factor recommended by manufacturer
CD57 BV605, Human, Flow cytometry, PBMCs, dilution factor recommended by manufacturer
CD122 (IL-2Rβ) PE, Human, Flow cytometry, PBMCs, dilution factor recommended by manufacturer
CD10 BUV395, Human, Flow cytometry, Peripheral blood, dilution factor recommended by manufacturer
CD13 PE-Cy7, Human, Flow cytometry, Peripheral blood, dilution factor recommended by manufacturer
CD14 BV650, Human, Flow cytometry, Peripheral blood, dilution factor recommended by manufacturer
CD15 BV605, Human, Flow cytometry, Peripheral blood, dilution factor tested in house
CD16 PerCP-Cy5.5, Human, Flow cytometry, Peripheral blood, dilution factor recommended by manufacturer
CD33 BV711, Human, Flow cytometry, Peripheral blood, dilution factor recommended by manufacturer
CD62L PE-CF594, Human, Flow cytometry, Peripheral blood, dilution factor recommended by manufacturer
CD123 BV786, Human, Flow cytometry, PBMCs, dilution factor recommended by manufacturer
CD185 Alexa700/APC-Cy5.5, Human, Flow cytometry, PBMCs, dilution factor recommended by manufacturer
CD203c BV421, Human, Flow cytometry, Peripheral blood, dilution factor recommended by manufacturer
CD279 FITC, Human, Flow cytometry, PBMCs stimulated with PHA, dilution factor recommended by manufacturer
CD64 APC-H7, Human, Flow cytometry, Peripheral blood, dilution factor tested in house
CD303 APC/Alexa647, Human, Flow cytometry, PBMCs, tested in house
CD83 PE-Cy5, Human, Flow cytometry, PBMCs stimulated with GM-CSF+IL-4+TNF –α, dilution factor recommended by manufacturer

CD172b PE, Human, Flow cytometry, Peripheral blood, dilution factor recommended by manufacturer

Immunohistochemistry (Antibody, Application, Sample used for validation, Dilution factor used for validation):
Universal mouse isotype, IHC-P, placenta tissue spiked with SARS-CoV-2 inactivated viruses, pre-made antibody/no dilution needed
Universal rabbit isotype, IHC-P, placenta tissue spiked with SARS-CoV-2 inactivated viruses, pre-made antibody/no dilution needed
SARS-CoV-2 (COVID-19) spike, IHC-P, placenta tissue spiked with SARS-CoV-2 inactivated viruses, dilution range recommended by manufacturer and dilution factor tested in house
SARS-CoV-2 (COVID-19) nucleocapsid, IHC-P, placenta tissue spiked with SARS-CoV-2 inactivated viruses, dilution range recommended by manufacturer and dilution factor tested in house

Human research participants

Policy information about studies involving human research participants

Population characteristics	The total study population included pregnant women ranging from 19 - 37 years of age (mean age 27.4 years). All patients self-identified as African-American or White. There were no statistical differences in relevant covariates (age, BMI, primiparity, mode of delivery, etc.) between the study and control groups.
Recruitment	Patients were recruited from among all pregnant women who presented at DMC Hutzel Women's Hospital in Detroit, Michigan, USA. Outside of SARS-CoV-2 positivity, there was no bias in the selection of patients for recruitment. All recruited patients self-identified as African-American, and thus our study population is largely representative of the general Detroit population.
Ethics oversight	The collection and use of human materials for research purposes were approved by the Institutional Review Boards of Wayne State University School of Medicine, Detroit Medical Center, and NICHD.

Note that full information on the approval of the study protocol must also be provided in the manuscript.

Flow Cytometry

Plots

Confirm that:

- [x] The axis labels state the marker and fluorochrome used (e.g. CD4-FITC).
- [x] The axis scales are clearly visible. Include numbers along axes only for bottom left plot of group (a 'group' is an analysis of identical markers).
- [x] All plots are contour plots with outliers or pseudocolor plots.
- [x] A numerical value for number of cells or percentage (with statistics) is provided.

Methodology

Sample preparation	Whole maternal peripheral blood and umbilical cord blood were used for flow cytometry without additional processing.
Instrument	BD LSRFortessa (BD Biosciences)
Software	Flow cytometry data was collected using FACSDiva version 6.0 (BD Biosciences). Flow cytometry data analysis was performed using FlowJo version 10 (FlowJo).
Cell population abundance	Cell sorting to purify specific cell populations for downstream applications was not performed.
Gating strategy	FSC/SSC gating was set to include the main leukocyte population and exclude debris. Positivity thresholds were determined using isotype antibodies and unstained (autofluorescence) controls.

- [x] Tick this box to confirm that a figure exemplifying the gating strategy is provided in the Supplementary Information.
